# In vivo assessment of the passive stretching response of the bicompartmental human semitendinosus muscle using shear-wave elastography

**DOI:** 10.1152/japplphysiol.00473.2021

**Published:** 2021-12-23

**Authors:** Adam Kositsky, David J. Saxby, Kim J. Lesch, Rod S. Barrett, Heikki Kröger, Olli Lahtinen, Laura E. Diamond, Rami K. Korhonen, Lauri Stenroth

**Affiliations:** ^1^Griffith Centre of Biomedical and Rehabilitation Engineering (GCORE), Menzies Health Institute Queensland, Griffith University, Gold Coast, Queensland, Australia; ^2^Department of Applied Physics, University of Eastern Finland, Kuopio, Finland; ^3^Sports and Exercise Medicine, Institute of Biomedicine, School of Medicine, University of Eastern Finland, Kuopio, Finland; ^4^Department of Orthopaedics, Traumatology and Hand Surgery, Kuopio University Hospital, Kuopio, Finland; ^5^Kuopio Musculoskeletal Research Unit (KMRU), Institute of Clinical Medicine, School of Medicine, University of Eastern Finland, Kuopio, Finland; ^6^Diagnostic Imaging Centre, Department of Clinical Radiology, Kuopio University Hospital, Kuopio, Finland; ^7^Unit of Clinical Radiology, Institute of Clinical Medicine, School of Medicine, University of Eastern Finland, Kuopio, Finland

**Keywords:** force, hamstrings, mechanical properties, musculoskeletal modeling, slack angle

## Abstract

The semitendinosus muscle contains distinct proximal and distal compartments arranged anatomically in series but separated by a tendinous inscription, with each compartment innervated by separate nerve branches. Although extensively investigated in other mammals, compartment-specific mechanical properties within the human semitendinosus have scarcely been assessed in vivo. Experimental data obtained during muscle-tendon unit stretching (e.g., slack angle) can also be used to validate and/or improve musculoskeletal model estimates of semitendinosus muscle force. The purpose of this study was to investigate the passive stretching response of proximal and distal human semitendinosus compartments to distal (knee) joint extension. Using two-dimensional shear-wave elastography, we bilaterally obtained shear moduli of both semitendinosus compartments from 14 prone-positioned individuals at 10 knee flexion angles [from 90° to 0° (full extension) at 10° intervals]. Passive muscle mechanical characteristics (slack angle, slack shear modulus, and the slope of the increase in shear modulus) were determined for each semitendinosus compartment by fitting a piecewise exponential model to the shear modulus-joint angle data. We found no differences between compartments or legs for slack angle, slack shear modulus, or the slope of the increase in shear modulus. We also found that the experimentally determined slack angle occurred at ∼15°–80° higher knee flexion angles compared with estimates from two commonly used musculoskeletal models, depending on participant and model used. Overall, these findings demonstrate that passive shear modulus-joint angle curves do not differ between proximal and distal human semitendinosus compartments and provide experimental data to improve semitendinosus force estimates derived from musculoskeletal models.

**NEW & NOTEWORTHY** We conducted an elastography-based investigation of the passive stretching response of the proximal and distal compartments of the human semitendinosus muscle and found no difference in shear modulus-joint angle curves between compartments. We also found that common musculoskeletal models tend to misestimate semitendinosus slack angle, most likely due to typical model assumptions. These results provide an important step toward a better understanding of semitendinosus passive muscle mechanics and improving computational estimates of muscle force.

## Graphical abstract

**Figure:**
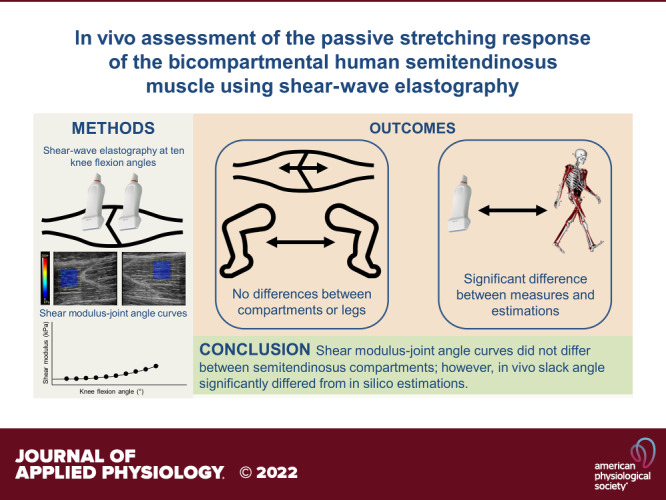

## INTRODUCTION

The semitendinosus (ST) is a fusiform hamstring muscle comprised of proximal (ST_prox_) and distal (ST_dist_) compartments separated by a band of connective tissue, termed the tendinous inscription, that commences at around one-third of muscle length and courses obliquely into the lower half of the muscle (1–3). Despite each compartment containing separate nerve branch innervations, muscle fibers in both compartments are oriented in parallel and nearly all fibers originate or terminate on the tendinous inscription, indicating an anatomically in-series arrangement of ST compartments (1, 4). The role of this tendinous inscription is unclear and may be an ontogenetic amalgamation of two different muscles or muscle heads (5, 6), a junction to connect in-series muscle fibers/fascicles (7), and/or serve to buffer forces and strains between these serially connecting muscle fibers/fascicles (8). Despite lingering questions regarding the function of the tendinous inscription, extensive investigations have revealed that ST_prox_ and ST_dist_ are mechanically in series in cats (4, 9, 10). However, mechanical and material muscle properties are variable between vertebrate species (11–13). For example, the maximum single fiber and fiber bundle tangent moduli are ∼20–25 times greater in the frog compared with human ST muscle (13, 14). Furthermore, it has been established that neuromuscular properties and behavior observed in cats do not always extend to humans (15, 16) and thus observations made from cat ST must be empirically confirmed in human ST. Studies clearly distinguishing human ST_prox_ and ST_dist_ have been limited to measuring intramuscular electromyographic amplitudes during isometric contractions (17) and architectural and morphological measures from cadavers (1, 8, 18) or children with and without spasticity (19, 20). As none of these previous studies directly quantified forces or stiffness within each compartment, further information is required to adequately assess the mechanical properties of human ST compartments.

Some mechanical properties of human ST compartments can be ascertained noninvasively using two-dimensional shear-wave elastography (2D-SWE), an ultrasound imaging technique quantifying Young’s modulus and/or shear modulus. The strong linear relationship between muscle shear modulus obtained with 2D-SWE and force output allows shear modulus measures to be used as an index of the change in muscle force (21, 22). Due to technical limitations (e.g., elastography map saturation; see Refs. 23 and 24), shear modulus cannot currently be assessed in the hamstrings in vivo at maximal levels of muscle activation. Conversely, passive ST shear modulus has been assessed in several studies, during both static resting (25–27) and passive stretching (28, 29); however, no 2D-SWE studies have thoroughly examined both ST compartments in humans. Furthermore, the joint angle or musculotendinous length at which ST begins to produce passive tension (i.e., slack angle or slack length, respectively) were not quantified in previous studies, where participants’ hips were highly flexed, likely because shear modulus seemingly increased immediately after stretch onset (see Fig. 5*A* of Ref. 28 and Fig. 2 of Ref. 29). Recently, Miyamoto et al. (27) found the resting shear modulus of hamstring muscles to be nonuniformly distributed proximodistally; however, ST compartments were not mentioned and, as measures were only obtained at four joint configurations, it remains unknown where in the range of motion passive force begins to increase in both compartments. Therefore, more detailed analyses of the passive stretching response (slack angle, slope of increase in tension, etc.) are required to better characterize and compare ST compartmental mechanical properties in humans. Furthermore, despite no between-leg asymmetry in shear modulus at two knee joint angles in uninjured individuals (26), it would be of importance to confirm that this symmetry also holds for parameters characterizing the passive stretching response to establish a baseline for potential future between-leg comparisons (e.g., previous hamstring strain injury, after anterior cruciate ligament reconstruction, etc.).

The in vivo assessment of the passive stretching response also provides important information for in silico muscle models. Most biomechanical muscle models, which are used to noninvasively estimate muscle forces due to the invasive nature of direct force measurements (30), consider passive tension to commence at the optimal fiber length (i.e., the point of maximal isometric force; see Refs. 31–33). As such, a muscle’s slack length, along with its optimal fiber length, determines the joint angles over which the respective muscle produces active and passive forces in muscle models (31, 34). However, experimental data demonstrate that optimal length and slack length are generally not interchangeable (12, 35–39), causing errors in passive muscle force estimations (35). Accurate modeling of passive force generation in muscle is particularly important when studying clinical populations with spasticity (e.g., cerebral palsy, hemiplegia, etc.) (40, 41) or simulating surgical effects on function (e.g., tendon transfer surgeries, osteotomy, etc.) (34). Furthermore, when studying healthy populations (e.g., athletes), incorrect estimations of passive muscle force may influence estimations of total muscle force and energy cost in individual muscles during dynamic tasks that include the full range of motion (e.g., hamstring muscles during sprinting, triceps surae and quadriceps muscles during stretch-shortening cycle tasks, etc.), where large active and passive forces are present at long muscle-tendon unit lengths. In addition, common musculoskeletal models (34, 42) apply identical parameters for a given muscle across both limbs and represent ST as a single muscle, based on the assumption that the two compartments are also mechanically in series in humans (43, 44). Therefore, bilateral in vivo experimental determination of slack angle in ST_prox_ and ST_dist_ would help inform and/or validate current commonly used musculoskeletal/biomechanical models.

The aims of this study were to compare *1*) passive shear modulus-joint angle curves of ST_prox_ and ST_dist_ in response to muscle-tendon unit lengthening, and *2*) experimentally determined slack angles with slack angles estimated from common musculoskeletal models. Based on the results of animal studies (e.g., 4, 9, 35, 36), it was hypothesized that shear modulus-joint angle curves would not differ between ST_prox_ and ST_dist_ in either leg, and that subject-specific experimental measures of ST slack angle would differ from in silico estimates. It was also hypothesized that shear modulus-joint angle curves would not differ between legs.

## MATERIALS AND METHODS

### Participants

Fourteen individuals (three females, age: 28.3 ± 4.4 yr, height: 176.1 ± 5.4 cm, mass: 72.0 ± 11.4 kg) volunteered to participate. Measures were obtained bilaterally; however, three participants had a prior anterior cruciate ligament reconstruction with an ipsilateral ST tendon graft on their nondominant leg. Data from the anterior cruciate ligament reconstructed legs were unused; however, data from the contralateral, uninjured legs were included as ST morphology in these legs has been shown not to differ compared with presurgical (45) and control group (46) measures. Thus, data are presented from 14 dominant and 11 nondominant legs. Potential participants were deemed ineligible if they had previous knee surgeries (other than anterior cruciate ligament reconstruction), acute muscle injuries, a body mass index <18 or >35 kg·m^−2^, or any cardiorespiratory or neurological diseases. Participants were requested to refrain from strenuous exercise commencing 24 h before data collection. The study was approved by the Research Ethics Committee of the Northern Savo Hospital District (348/2020) and adhered to the Declaration of Helsinki. All participants provided written informed consent before participation.

### Experimental Procedure

No warm-up was provided to avoid any confounding effect on hamstring shear moduli (25). Participants were positioned prone throughout the entire experiment. The hip was not fixed to the plinth; however, the hip hiking generally observed when participants perform active contractions was not visually observed when passively rotating through the range of motion. For preconditioning purposes, the knee joint was manually rotated slowly and passively through the range of motion five times by an investigator. Then, 2D-SWE images (see *Shear-Wave Elastography Measurements* section for details) were obtained at 10° intervals from 90° of knee flexion until full knee extension (0°) was reached, with at least 10 s rest given between changing knee position and subsequent image acquisition. The leg was held manually by an investigator at all angles except 0°, where the leg was resting along the table. Knee joint angle was verified and monitored in real-time by a calibrated electrogoniometer (ME6000, Bittium, Finland). When bilateral measurements were made, the left leg was measured first.

Surface electromyograms (ME6000, Bittium, Finland) were recorded from the semimembranosus muscle with bipolar Ag/AgCl electrodes (Ambu Blue Sensor N-00-S, Ambu A/S, Ballerup, Denmark) placed in the direction of muscle fascicles under ultrasound guidance and with an interelectrode distance of 22 mm. As it was not possible to simultaneously acquire elastograms and electromyograms from ST compartments, the semimembranosus muscle was chosen to represent the hamstring group as its muscle belly is more distal on the thigh (47) and thus electrodes would not interfere with positioning the ultrasound transducer over ST. Electromyography feedback was provided in real-time to both examiners and participants to ensure myoelectric silence during image acquisition.

### Shear-Wave Elastography Measurements

**Figure 1.**
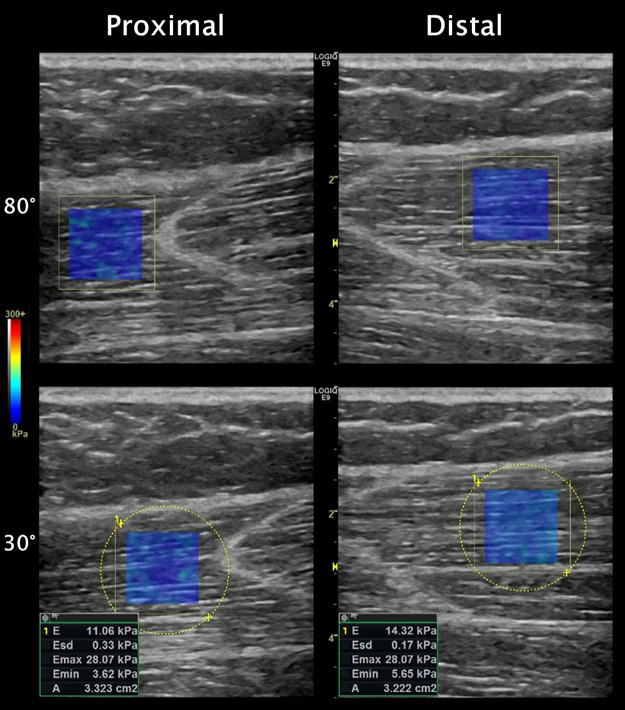
Exemplary shear-wave elastography images of the proximal (*left*) and distal (*right*) compartments of semitendinosus at 80° (*upper*) and 30° (*lower*) of knee joint flexion. The colored rectangle within each ultrasound image is the elastography map, with the scale (Young’s modulus) on the *outer left* of the figure. Note the similarities between ultrasound image features despite repositioning the transducer over the skin for every measurement (without the aid of indelible ink markings). The tendinous inscription is visible as the hyperechoic V-shaped structure adjacent to the elastography color maps. Examples of the measurement method using the internal software of the device are shown on the lower images.

A full description of 2D-SWE is found in detail elsewhere (21, 48, 49). Briefly, the ultrasound elastography device (LOGIQ E9, GE Healthcare, Wauwatosa, WI) calculates the tissue’s Young’s modulus (*E*) for each pixel within a given region of interest by the formula:

(*1*) $$E=3\rho c_{t}^{2}$$

where ρ is tissue density (assumed to be 1,000 kg**^.^**m^−3^) and *c_t_* is the shear-wave speed (m**^.^**s^−1^). Elastography maps of ST_prox_ and ST_dist_ were obtained from images acquired using a 44-mm linear transducer (9L; operating frequency: 8.4 MHz; field-of-view depth: 5 to 6 cm). The elastography color map was scaled to the maximum capability of the device to be able to clearly see any potential artifacts within the map. The transducer was carefully aligned along the fascicle plane and held manually with minimal pressure over the skin (28). Transducer placement along the leg was not standardized due to interindividual variation in tendinous inscription location and substantial shifting of the ST muscle under the skin with changes in knee joint angle. Rather, at each joint angle, the transducer was placed close to the tendinous inscription, and the positioning was deemed appropriate when fascicles could be clearly seen throughout the image (Fig. 1). At any given joint angle, images were acquired from ST_prox_ and ST_dist_ in alternating fashion until three acceptable images were obtained from each compartment. Images were saved after allowing a few seconds for the elastography map to stabilize. Acoustic coupling gel was applied to prevent tissue compression, and the transducer was replaced over the skin for each image acquisition. The elastography map size was held constant at ∼1.15 cm × ∼1.15 cm, and the same investigator (A.K.) acquired all images.

Measurements were not conducted/completed for ST_prox_ of both legs of one participant and the dominant leg of another participant due to poor visibility of ST_prox_ and/or excessive voids in the elastography map. Thus, images were obtained for *n* = 12 (dominant) and *n* = 10 (nondominant) for ST_prox_, and *n* = 14 (dominant) and *n* = 11 (nondominant) for ST_dist_. In addition, data were excluded for ST_dist_ at 80° from one nondominant leg due to errors in the recordings, but subsequent analysis of the shear modulus-joint angle curve (see *Data Analyses* section) for this compartment was not affected.

### Data Analyses

All elastography images were analyzed using the internal software of the ultrasound device. For each image, the mean Young’s modulus value was obtained by placing a circular area around the rectangular elastography map (Fig. 1). All pixels outside or voids within the map were automatically excluded from measurements. The “Emax” output of every image was checked to ensure no pixels within the elastography map were saturated at 300 kPa (i.e., exceeded the threshold of the device). The mean Young’s modulus values were divided by three to obtain shear modulus, which, compared with Young’s modulus, is a more appropriate 2D-SWE measure for anisotropic soft tissues such as muscle (21, 48, 49). The standard error of measurement of shear moduli measures was 5.9%–14.4%. Full measurement reliability and potential effects on interpretation are described in the supplemental material (Supplemental Table S1 and Fig. S1; see https://doi.org/10.6084/m9.figshare.16837417).

A piecewise exponential model describing the passive length-tension relationship of skeletal muscle was then fitted to the data (50):

(*2*) $$\begin{array}{l} G\left(\theta \right)=G_{O}\;\;if\;\theta \leq \theta_{O}\\ G\left(\theta \right)=G_{O}\left(e^{\alpha \left(\theta -\theta_{O}\right)}\right)\;\;\;\;\;if\;\theta >\theta_{O} \end{array}$$

where G(θ) is the shear modulus at any given knee joint angle, G_o_ and θ_o_ are the shear modulus and knee joint angle, respectively, at which passive tension begins to develop (i.e., slack angle), and α is an exponential constant defining the slope of the increase in passive tension.

**Figure 2.**
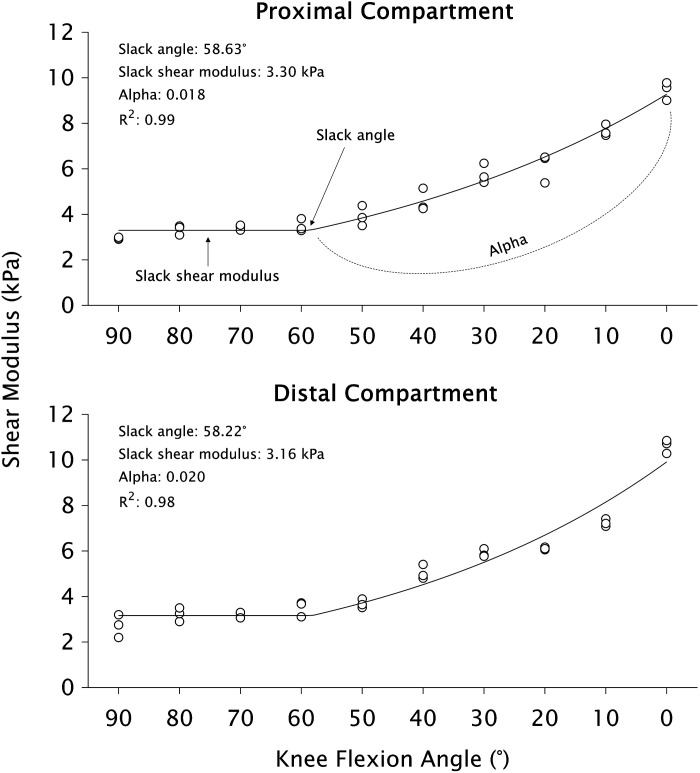
Representative data of the piecewise exponential model fitted to 10 knee joint angles of the proximal (*upper*) and distal (*lower*) semitendinosus compartments for one participant. Circles represent individual trial measures at each knee flexion angle (0° = full knee extension). The solid line represents the curve fit.

The values of θ_o_, G_o_, and α and were optimized using custom-made scripts in MATLAB (version R2018b, MathWorks, Natick, MA) by minimizing the least square difference between experimentally measured and modeled shear moduli using the Levenberg–Marquardt algorithm (Fig. 2). The default starting search values for the optimization were 50° (θ_o_), 3 kPa (G_o_), and 0.01 (α), with no boundaries. Although the initial search values would not affect the results, we elected to apply consistent values (i.e., across all legs and compartments) chosen based on initial analyses of the entire data set. The curve was fitted to the mean shear modulus value from the three images at each knee joint angle. We observed that initial decreases in shear modulus sometimes occurred at very short muscle lengths (e.g., Fig. 3*A* of Ref. 51), which would affect the output of the piecewise model. Thus, if the shear moduli values at 90° and 80° did not overlap when the minimal detectable change (see Supplemental material) was accounted for, the data associated with 90° was removed from the curve-fitting model (*n* = 2 legs). The coefficient of determination (*R*^2^) of the fit between experimental and modeled data was also calculated. The curve-fit was accepted when θ_o_ ≤ 85° and *R*^2^ ≥ 0.85.

The Rajagopal (42) and gait2392 (based on Ref. 34) musculoskeletal models were loaded separately in OpenSim (version 3.3, SimTK, Stanford, CA). Activation of the right ST was set at 0.01 and the hip flexion joint angle locked at 0°. Data from the passive fiber-force—knee joint angle curve were then extracted for every ∼1.21° (Rajagopal) or ∼1.31° (gait2392) of the right knee joint angle. The first joint angle where force was above zero was deemed the model-estimated θ_o_. As force-angle curves (i.e., operating range) are typically made generic across participants (52), only the default models were used.

### Statistical Analyses

Full-factorial linear mixed models with restricted maximum likelihood estimation were performed to assess the effects of leg (dominant, nondominant) and compartment (proximal, distal) on slack angle, slack shear modulus, and α. Leg and compartment were set as fixed factors and repeated measures (diagonal covariance structure), with intercepts for participants as a random factor. The degrees of freedom were calculated using the Satterthwaite approximation. Post hoc Bonferroni tests were conducted when significant interactions occurred. The experimentally determined slack angle (averaged across all compartments and legs for each participant) was compared with musculoskeletal model values using a one-sample *t* test. All hypothesis testing was conducted using SPSS (version 27, SPSS Inc., Chicago, IL) with statistical significance set at *P* < 0.05. Data are presented as means ± standard deviation.

## RESULTS

**Figure 3.**
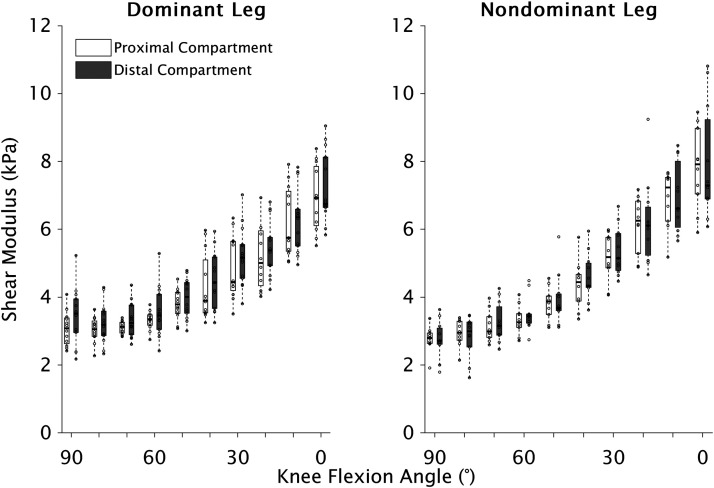
Boxplots of shear modulus values of proximal (unfilled) and distal (filled gray) semitendinosus compartments obtained at 10 knee flexion angles (0° = full knee extension) of dominant (*left*) and nondominant (*right*) legs. For ST_prox_, data are presented for dominant (*n* = 12) and nondominant (*n* = 10) legs, and dominant (*n* = 14) and nondominant (*n* = 11) legs for ST_dist_ (except for 80°, where *n* = 10; see methods section for details). ST, semitendinosus; ST_prox_, ST proximal; ST_dist_, ST distal.

**Figure 4.**
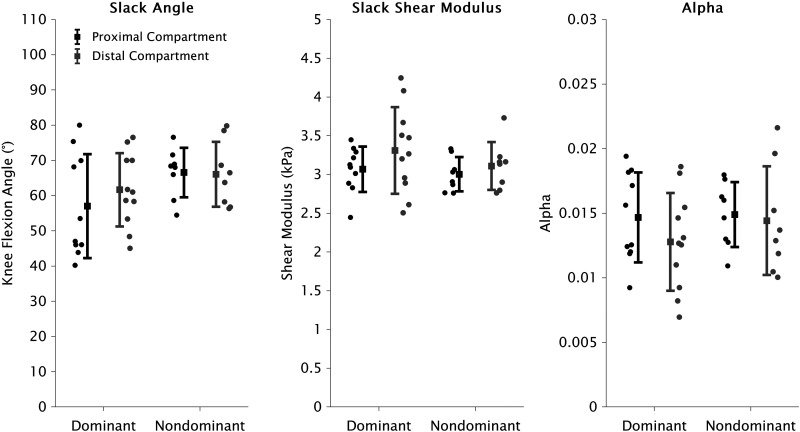
Means and standard deviations of slack angle (*left*), slack shear modulus (*center*), and the slope of the increase in shear modulus (α) (*right*) of proximal (black) and distal (gray) semitendinosus compartments of dominant and nondominant legs. Filled circles represent individual datapoints. For ST_prox_, data are presented for dominant (*n* = 10) and nondominant (*n* = 8) legs, and dominant (*n* = 11) and nondominant (*n* = 8) legs for ST_dist_. Linear mixed models found no main effects or interactions of leg and/or compartment for slack angle, slack shear modulus, or α. ST, semitendinosus; ST_prox_, ST proximal; ST_dist_, ST distal.

Shear modulus-joint angle data are summarized in Fig. 3. The piecewise model (*Eq. 2*) was only accepted for 37/47 (79%) tested compartments, with all individual data available in supplemental material. There were no main effects of leg or compartment for slack angle [leg: *F*(1,23.96) = 4.25, *P* = 0.050; compartment: *F*(1,19.97) = 0.57, *P* = 0.458], slack shear modulus [leg: *F*(1,24.51) = 1.20, *P* = 0.285; compartment: *F*(1,21.19) = 2.47, *P* = 0.131], or α [leg: *F*(1,24.63) = 0.48, *P* = 0.494; compartment: *F*(1,20.91) = 1.77, *P* = 0.198] (Fig. 4). There were also no interactions between leg and compartment for slack angle [*F*(1,19.23) = 0.66, *P* = 0.426], slack shear modulus [*F*(1,20.35) = 0.40, *P* = 0.532], or α [*F*(1,20.06) = 0.95, *P* = 0.341].

**Figure 5.**
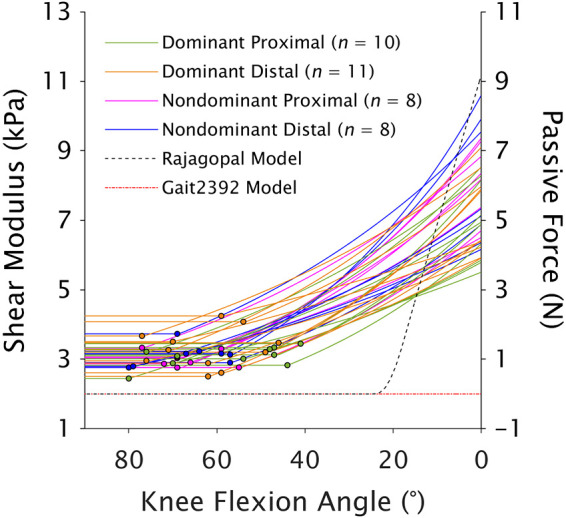
Comparison of experimental measures of semitendinosus slack angle with estimates from musculoskeletal models (32, 40), where 0° of knee flexion angle represents full knee extension. Filled circles superimposed over experimental data lines represent the experimentally determined slack angle for each participant/leg/compartment. The *left y*-axis (shear modulus) corresponds with the experimental data, and the *right y*-axis (passive force) corresponds with the generic in silico data. Note there are three overlapping circles at ∼69° of knee flexion. The slack angle determined with shear-wave elastography was significantly different (*P* < 0.001; one-sample *t* test) than the slack angle estimated from both models.

The slack angle was 24.24° of knee flexion for the Rajagopal model and 2.12° of knee (hyper)extension for the gait2392 model (Fig. 5). The experimentally determined slack angle (62.31 ± 8.94°) occurred at significantly larger knee flexion angles compared with the Rajagopal (*t* = 15.36, *P* < 0.001) and gait2392 (*t* = 25.99, *P* < 0.001) models.

## DISCUSSION

This study investigated the passive stretching response of two anatomically in-series muscle compartments, the proximal and distal compartments of the human ST muscle, in vivo using 2D-SWE. Consistent with our hypotheses, parameters from shear modulus-joint angle curves were not significantly different between ST compartments, and the experimentally observed slack angle occurred at significantly higher knee flexion angles compared with in silico estimates. These findings suggest that passive mechanical properties do not differ between the two ST compartments and provide experimental data for improving force estimates from musculoskeletal models.

### Semitendinosus Shear Modulus-Joint Angle Curves between Legs and Muscle Compartments

In agreement with previous elastography studies demonstrating no difference in active (23) or passive (26) ST shear moduli between dominant and nondominant legs, no measures from the piecewise model (θ_o_, G_o_, or α) differed between legs. Passive shear modulus-joint angle measures also did not differ between ST compartments, which is to an extent analogous with Lacourpaille et al. (53), who reported no difference in slack angle between the two heads of the biceps brachii. Conversely, Le Sant et al. (54) found slack angle to differ proximodistally within soleus, but not medial or lateral gastrocnemius, potentially due to the complex four-compartment structure of soleus (55). Despite its tendinous inscription, ST may be considered simpler than soleus as ST_prox_ and ST_dist_ are parallel-fibered and anatomically in series. Thus, given the linear relationship between the change in shear modulus and the change in passive force (21, 22), the lack of differences in slack angle, slack shear modulus, and α (i.e., exponential increase in shear modulus) between ST_prox_ and ST_dist_ suggests that forces are also not different throughout the entire prone range of motion between these two anatomically in-series ST compartments, at least during passive stretching. Whether forces in each compartment are independently but equally developed simultaneously or shared/balanced across compartments cannot be directly answered from the current experiment; however, some inferences may be suggested based on the results of previous studies. Stimulating the nerve branch innervating the cat ST_prox_ resulted in similar force recorded at the distal ST tendon compared with both ST_dist_ nerve branch stimulation and simultaneous stimulation of both ST_prox_ and ST_dist_ (4), and passive force development was greater in distal compared with proximal regions of the pennate rat extensor digitorum longus when the muscle was stretched distally (38). Furthermore, as the muscles directly adjacent to ST (biceps femoris long head and semimembranosus) are also biarticular hip extensors and knee flexors (43), any epimuscular myofascial force transmission affecting proximodistal distribution of muscle force should be minimal given this phenomenon is more likely to occur when adjacent muscles are displaced relative to one another (38, 56). Therefore, the potential for force transmission from one compartment to the other to equilibrate force across the whole human ST muscle, rather than simultaneously equal independent force development, must be strongly considered.

Although shear moduli, and thus forces, may not differ between ST compartments, the same may not hold true for stresses and strains. Unfortunately, absolute or relative length changes could not be quantified from our ultrasound images as ST fascicle length exceeds the transducer’s field of view and the parallel fascicle architecture does not allow for extrapolation methods. We have, however, qualitatively observed greater movement of fascicles in ST_dist_ compared with ST_prox_ throughout the range of motion that was examined in the present study, which is consistent with previous studies reporting distal sarcomeres (57) and fascicles (58) of other lower limb muscles lengthen more than their proximal counterparts in response to distal joint lengthening. Considering that the physiological cross-sectional area of cadaveric ST_prox_ is significantly greater than ST_dist_ (18), larger strains in ST_dist_ during passive knee extension might be expected for a given shear modulus and force, as stress (force per area) would also likely be larger. Likewise, unequal strains between ST_prox_ and ST_dist_ at similar force levels have been reported in cats (4). Thus, it appears there may be a trade-off in that balancing moduli and force across two in-series compartments may result in disproportionate stresses and strains imposed on each compartment, which would have implications for musculotendinous injuries (4) but requires further investigation. Future studies that combine 2D-SWE with ultrasound strain imaging (59, 60) and/or microendoscopy (61) to concomitantly measure mechanical properties and length changes within each compartment are therefore recommended.

### In Vivo and In Silico Slack Angles

The wide range in individual ST slack angles (∼40°–80°) in the present study is comparable with interindividual variation in slack angle of other thigh muscles (51, 62). Interestingly, ST slack angle occurred at relatively high knee flexion angles (i.e., close to the shortest in vivo muscle length) in some participants. Recently, Kaya et al. (63) reported passive force-angle curves of spastic ST obtained intraoperatively with a buckle transducer attached to the distal tendon. Although direct comparisons are not straightforward owing to different demographic groups and methods, their data show some individuals, based on the standard deviation around the mean, developed passive ST forces even at 90° of knee flexion (hip flexion = 20°; Fig. 3*B* of Ref. 63). This demonstrates ST slack angle at high knee flexion angles in some healthy participants is plausible, although invasive measures would be required for verification.

Given the mechanical properties did not differ between ST_prox_ and ST_dist_, modeling the human ST as one whole muscle may be justified when estimating global muscle forces and joint contact forces in healthy, nonfatigued individuals. However, the experimentally determined ST slack angle systematically (i.e., for all compartments and legs) occurred at much deeper knee flexion angles compared with the commonly used Rajagopal (42) and gait2392 (based on Ref. 34) musculoskeletal models (difference of ∼15°–80°, depending on participant and model used; Fig. 5). The discrepancy in slack angle may be due to errors in muscle-tendon unit length estimations in musculoskeletal models (64), but more likely originates from assumptions in biomechanical muscle models, which typically set passive force onset at optimal fiber length (31–33). Although ST sarcomere length is close to optimal length in the anatomical position (18, 43, 65) and thus ST optimal fiber length is likely set correctly in muscle models, the main source of passive tension in whole muscles stems from extracellular matrix structures and not from the sarcomere (66, 67). Accordingly, experimental data have shown slack length is generally not synonymous with optimal length nor standardized across muscles (12, 35–39), leading to inaccurate predictions of passive forces when assuming passive force onset at optimal fiber length (35). Although passive ST forces through the knee range of motion in the prone position are small, due to the exponential shape of the passive tension curve, total forces toward the end of the range of motion (i.e., with the hip flexed) can be severely misestimated as small length changes at long lengths cause large differences in passive forces (68). Given the discrepancy between experimental and modeled ST slack angle, this assumption may have influenced estimations of force distribution within the hamstrings during, for example, terminal swing phase during running (69, 70) by incorrectly allocating individual muscle forces that summate to match the measured joint accelerations/moments. The joint angle at which ST slack angle occurred, coupled with the interindividual variability, suggests accurate muscle force estimates may require subject-specific muscle measures and adjusting the passive force-length curve, as done by Lloyd and Besier (71), to allow passive force generation below optimal fiber length. More complex models (e.g., finite element methods) may be needed to assess localized and compartment stresses and strains within ST, though this was not the target of the current study and should be investigated in future studies.

### Methodological Considerations and Study Limitations

The piecewise model was accepted for only 79% of the tested ST compartments. Data not accepted did not appear to have a plateau region in shear modulus-joint angle curves (see Supplemental data plots) and were generally from both compartments of a given leg. Thus, parameters from compartments with θ_o_ ≥ 85° may indeed be accurate. Conversely, previous studies fitting a piecewise model to 2D-SWE data obtained passively had acquired shear moduli at ≤5° intervals and with a relatively larger plateau (slack) region (50, 72). Measuring at ≤5° intervals throughout a greater range of motion for the present study was not time feasible without conducting slow, passive stretches on an isokinetic dynamometer. However, we deemed it necessary to reposition the transducer between joint angles to maintain image location along the muscle (due to substantial sliding under the skin), optimize image quality, and reduce systematic measurement errors. Thus, it is possible there may have been a very narrow plateau region for these compartments had we measured at smaller joint angle intervals. Although we cannot confirm the accuracy of results where θ_o_ ≥ 85°, we reperformed the linear mixed model including all data with *R*^2^ ≥ 0.85. As the main results did not change (Supplemental Fig. S2), we are confident in our overall interpretation.

The following limitations should also be considered when interpreting the findings of the present study. First, although the leg was held manually at each joint angle rather than controlled by an isokinetic dynamometer, our absolute reliability measures were comparable with previous literature and additional analyses accounting for the measurement error did not affect the interpretation (Supplemental Table S1 and Fig. S1). Thus, the limitations of our experimental setup likely did not influence the results or our conclusions. Second, we only obtained passive measurements in the prone position. Previous studies depicting passive ST shear modulus-joint angle curves obtained in 70°–120° of hip flexion demonstrated an almost immediate increase in shear modulus, despite measurements commencing at 110°–120° of knee flexion (see Fig. 5*A* of Ref. 28 and Fig. 2 of Ref. 29). Therefore, placing the hip in its least flexed position was necessary for assessing ST slack angle. Given the α parameter, representing the exponential increase in shear modulus, did not differ between ST compartments, it is unlikely that hip flexion, where muscle length and thus shear modulus are both greater, would influence the findings. It should also be noted that the optimal sarcomere length of ST (as measured passively) is found within the prone range of knee motion (18, 43, 65). As a muscle’s optimum length is not considered to be a short muscle length and increases in shear modulus were found from the beginning to end of the prone range of motion in the present study (Fig. 3), even when accounting for the measurement error (Supplemental Fig. S1), our experimental design should not be considered insufficient for assessing ST slack angle. Third, images were obtained close to the tendinous inscription and not at different locations within each compartment; however, as the anatomical cross-sectional area of ST varies greatly along its length (47), confining data acquisition to a small region of the whole muscle reduced the effects anatomical cross-sectional area may have on the slope of the increase in the shear modulus-force relationship (22). In addition, image quality can be poor in more proximal portions of ST_prox_ (18, 20). Fourth, although we ensured electromyography silence of semimembranosus during data acquisition, we cannot definitively confirm ST was inactive as it was not possible to simultaneously acquire electromyograms and elastograms given *1*) the large gluteals covering ST_prox_ proximal to the transducer, and *2*) the available ST_dist_ electrode position being in the distal portion of the muscle, where ST is small compared with the adjacent synergists (47) and thus exposing the recordings to high amounts of cross talk. Nevertheless, given shear modulus-joint angle curves did not differ between compartments, any residual activation in ST may support the paradigm of force-sharing between compartments. Finally, the sample size did not allow for male and female subgroups; however, previous work found no between-sex differences in passive ST shear modulus (26) or slack angle of other lower limb muscles (50, 73).

### Conclusions

The passive stretching response did not differ between the anatomically in-series proximal and distal compartments of the human ST muscle. In addition, the experimentally determined slack angle occurred at larger knee flexion angles than slack angle estimates from commonly used musculoskeletal models. The overall findings of this study suggest the passive mechanical properties of proximal and distal ST compartments are not different and may be used to improve musculoskeletal model estimates of ST muscle force.

## SUPPLEMENTAL DATA

10.6084/m9.figshare.16837417 Supplemental Table S1 and Supplemental Figs. S1 and S2: https://doi.org/10.6084/m9.figshare.16837417.

## GRANTS

This work was supported in part by a Griffith University Postgraduate Research Scholarship and International Experience Incentive Scheme travel grant, the International Society of Biomechanics International Travel Grant program, the Erasmus+ Programme of the European Union, and the Academy of Finland (Grant No. 324529 and 332915).

## DISCLOSURES

No conflicts of interest, financial or otherwise, are declared by the authors.

## AUTHOR CONTRIBUTIONS

A.K., D.J.S., R.S.B., and L.S. conceived and designed research; A.K., K.J.L., and L.S. performed experiments; A.K. analyzed data; A.K., D.J.S., R.S.B., R.K.K., and L.S. interpreted results of experiments; A.K. prepared figures; A.K. drafted manuscript; A.K., D.J.S., K.J.L., R.S.B., H.K., O.L., L.E.D., R.K.K., and L.S. edited and revised manuscript; A.K., D.J.S., K.J.L., R.S.B., H.K., O.L., L.E.D., R.K.K., and L.S. approved final version of manuscript.
